# Complete chloroplast genome of a semi-mangrove plant *Hibiscus tiliaceus* (Malvaceae)

**DOI:** 10.1080/23802359.2021.1935337

**Published:** 2021-06-14

**Authors:** Gang Hu, Cong Hu, Xingbing Peng, Shaonuan Lu, Zhonghua Zhang

**Affiliations:** College of Environmental and Life Sciences, Nanning Normal University, Nanning, China

**Keywords:** *Hibiscus tiliaceus*, chloroplast genome, phylogenetic analysis

## Abstract

*Hibiscus tiliaceus* is a semi-mangrove species that is widely distributed in tropical and subtropical coastal areas around the world. Here, the complete chloroplast (cp) genome sequence of *H. tiliaceus* was assembled and characterized. The cp genome was 161,748 bp in length, consisting of a large single copy (LSC) region of 89,190 bp and a small single copy (SSC) region of 19,616 bp, which were separated by a pair of 26,471 bp inverted repeat (IR) regions. The overall GC content was 36.88%. A total of 131 genes, including 85 protein-coding genes, 37 tRNA genes and 8 rRNA genes were identified. Phylogenetic tree reconstructed by 15 complete cp genomes revealed that *H. tiliaceus* was sister to the congeneric species *H. cannabinus*.

*Hibiscus tiliaceus* L. (Malvaceae) is a common coastal semi-mangrove plant that is widely distributed in tropical and subtropical America, Africa, Asia, Australia, and throughout the Pacific islands (Abdul-Awal et al. 2016). As a fast-growing, wind- and salt-resistant tree or larger shrub, it grows well and is abundant in littoral forests and mangrove forest margins of atolls and high islands (Feng et al. 2008). It was used for the stabilization of sand dunes, the formation of coastal windbreaks, and also as a landscape tree species inland due to its wide adaptation various eco-geographic conditions (Tang et al. 2003). In addition, it has been used as a traditional medicine for the treatment of fevers and coughs, ear infections and abscesses, postpartum disorders, and skin diseases in Asian and African countries (Vanzella et al. 2012; Abdul-Awal et al. 2016). The phylogeography and genetic diversity of *H. tiliaceus* have been assessed on the basis of variations in chloroplast (cp) DNA sequences in several previous studies (Tang et al. 2003; Takayama et al. 2006; Yang et al. 2011; Shi et al. 2020). However, the information of the complete cp genome is still lacking. Here, we report and characterize the complete cp genome of *H. tiliaceus* to further study its genetic research and resource utilization.

Fresh leaves of *H. tiliaceus* were sampled from Beilun Gulf, Guangxi, China (N21°36′50″, E108°14′20″). The voucher specimen (NNU20200325) was stored in the Herbarium of Nanning Normal University. The complete genomic DNA was extracted from the fresh leaves using a modified CTAB protocol (Doyle and Doyle 1987). Complete genome sequencing was conducted by Genepioneer Biotechnologies Inc. (Nanjing, China) on the Illumina HiSeq platform. The filtered sequences were assembled using the program SPAdes assembler 3.10.0 (Bankevich et al. 2012). Annotation was performed using GeSeq (Tillich et al. 2017) and manually corrected.

The cp genome of *H. tiliaceus* was determined to comprise a 161,748 bp double-stranded, circular DNA (GenBank accession no. MT644160), containing two inverted repeat (IR) regions of 26,471 bp, separated by large single-copy (LSC) and small single-copy (SSC) regions of 89,190 and 19,616 bp, respectively. The overall GC content of the cp genome of *H. tiliaceus* is 36.88%. The cp genome was predicted to contain 131 genes, including 85 protein-coding genes, 37 tRNA genes, and 8 rRNA genes. Six protein-coding genes, seven tRNA genes, and four rRNA genes were duplicated in the IR regions. Fifteen genes contained one intron, while three had two introns (clpP, rps12, and ycf3). The corresponding values in IR, LSC, and SSC regions are 42.7%, 34.8, and 30.8, respectively.

To identify the phylogenetic position of *H. tiliaceus*, alignment was performed on the 15 cp genome sequences using MAFFT v7.307 (Katoh and Standley 2013), and a maximum likelihood (ML) tree was constructed by RAxML v8.2.10 (Stamatakis 2014). The results indicated that *H. tiliaceus* is mostly related to the congeneric species *H. cannabinus* (Figure 1). The complete cp genome sequence of *H. tiliaceus* will provide a useful resource for facilitating its genetic research and further utilization of *Hibiscus*.

**Figure 1.**
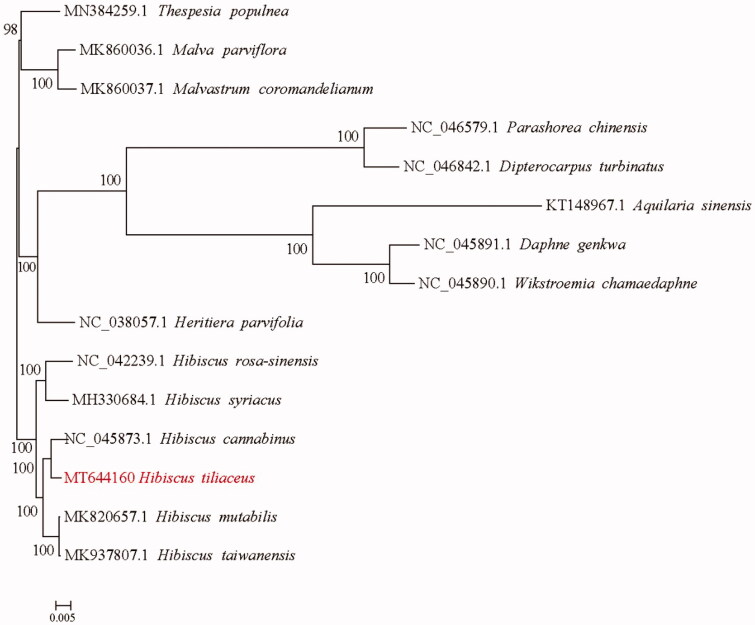
Phylogenetic relationship of the *H. tiliaceus* chloroplast genome with 14 previously reported complete chloroplast genomes. A total of 1000 bootstrap replicates were computed and the bootstrap support values are shown at the branches. GenBank accession numbers were shown.

## Data Availability

The genome sequence data that support the findings of this study are openly available in GenBank of NCBI at (https://www.ncbi.nlm.nih.gov/) under the accession no. MT644160. The associated BioProject, SRA, and Bio-Sample numbers are PRJNA666500, SRS7451015, and SAMN16295088, respectively.

## References

[CIT0001] Abdul-Awal SM, Nazmir S, Nasrin S, Nurunnabi TR, Uddin SJ. 2016. Evaluation of pharmacological activity of *Hibiscus tiliaceus*. Springerplus. 5(1):1209. 27516947 10.1186/s40064-016-2891-0 PMC4967069

[CIT0002] Bankevich A, Nurk S, Antipov D, Gurevich AA, Dvorkin M, Kulikov AS, Lesin VM, Nikolenko SI, Pham S, Prjibelski AD, et al. 2012. SPAdes: a new genome assembly algorithm and its applications to single-cell sequencing. J Comput Biol. 19(5):455–477. 22506599 10.1089/cmb.2012.0021 PMC3342519

[CIT0003] Doyle JJ, Doyle JL. 1987. A rapid DNA isolation procedure for small quantities of fresh leaf tissue. Phytochem Bull. 19:11–15.

[CIT0004] Feng C, Li XM, Ji NY, Wang BG. 2008. Triterpenoids from the mangrove plant *Hibiscus tiliaceus*. HCA. 91(5):850–855.

[CIT0005] Katoh K, Standley DM. 2013. MAFFT multiple sequence alignment software version 7: improvements in performance and usability. Mol Biol Evol. 30(4):772–780. 23329690 10.1093/molbev/mst010 PMC3603318

[CIT0006] Shi CC, Han K, Li LW, Seim I, Lee SMY, Xu X, Yang HM, Fan GY, Liu X. 2020. Complete chloroplast genomes of 14 mangroves: phylogenetic and comparative genomic analyses. Biomed Res Int. 2020:8731857. 32462024 10.1155/2020/8731857 PMC7225854

[CIT0007] Stamatakis A. 2014. RAxML version 8: a tool for phylogenetic analysis and post-analysis of large phylogenies. Bioinformatics. 30(9):1312–1313. 24451623 10.1093/bioinformatics/btu033 PMC3998144

[CIT0008] Takayama K, Kajita T, Murata J, Tateishi Y. 2006. Phylogeography and genetic structure of *Hibiscus tiliaceus*-speciation of a pantropical plant with sea-drifted seeds. Mol Ecol. 15(10):2871–2881. 16911207 10.1111/j.1365-294X.2006.02963.x

[CIT0009] Tang T, Zhong Y, Jian SG, Shi SH. 2003. Genetic diversity of *Hibiscus tiliaceus* (Malvaceae) in China assessed using AFLP markers. Ann Bot. 92(3):409–414. 12930729 10.1093/aob/mcg156 PMC4408444

[CIT0010] Tillich M, Lehwark P, Pellizzer T, Ulbricht-Jones ES, Fischer A, Bock R, Greiner S. 2017. GeSeq - versatile and accurate annotation of organelle genomes. Nucleic Acids Res. 45(W1):W6–W11. 28486635 10.1093/nar/gkx391 PMC5570176

[CIT0011] Vanzella C, Bianchetti P, Sbaraini S, Vanzin SI, Melecchi MIS, Caramão EB, Siqueira IR. 2012. Antidepressant-like effects of methanol extract of *Hibiscus tiliaceus* flowers in mice. BMC Complem Altern M. 12:41. 10.1186/1472-6882-12-41 PMC3407014 22494845

[CIT0012] Yang GL, Zhou RC, Tang T, Chen XS, Ouyang JH, He L, Li WJ, Chen SF, Guo MM, Li XN, et al. 2011. Gene expression profiles in response to salt stress in *Hibiscus tiliaceus*. Plant Mol Biol Rep. 29(3):609–617.

